# Isolation and Annotation of *Arthrobacter globiformis* Phage AWGoat

**DOI:** 10.17912/micropub.biology.001433

**Published:** 2025-01-16

**Authors:** Caitlyn Bolling, Chloe Chervenic, Kate Drake, Zoe Griffin, Isha Jain, Hala Khabir, Tyler Ordon, Anthony Padilla, Rachel Showers, Jose Vences, Caelan Walsh, Wyatt Workman, Sharon Bullock, Michelle Pass, Tonya Bates, Ellen Wisner

**Affiliations:** 1 Department of Biological Sciences, University of North Carolina at Charlotte

## Abstract

AWGoat, a lytic bacteriophage that infects *Arthrobacter globiformis,* was isolated from a goat pen in Charlotte, NC. Its genome is 65,496 bp long, with a GC content of 67.1%. Based on gene content similarity, AWGoat is assigned to actinobacteriophage cluster AP. It encodes a putative toxin from a broadly distributed toxin/antitoxin pair and an unusually large minor tail protein.

**Figure 1. The AWGoat genome map:**
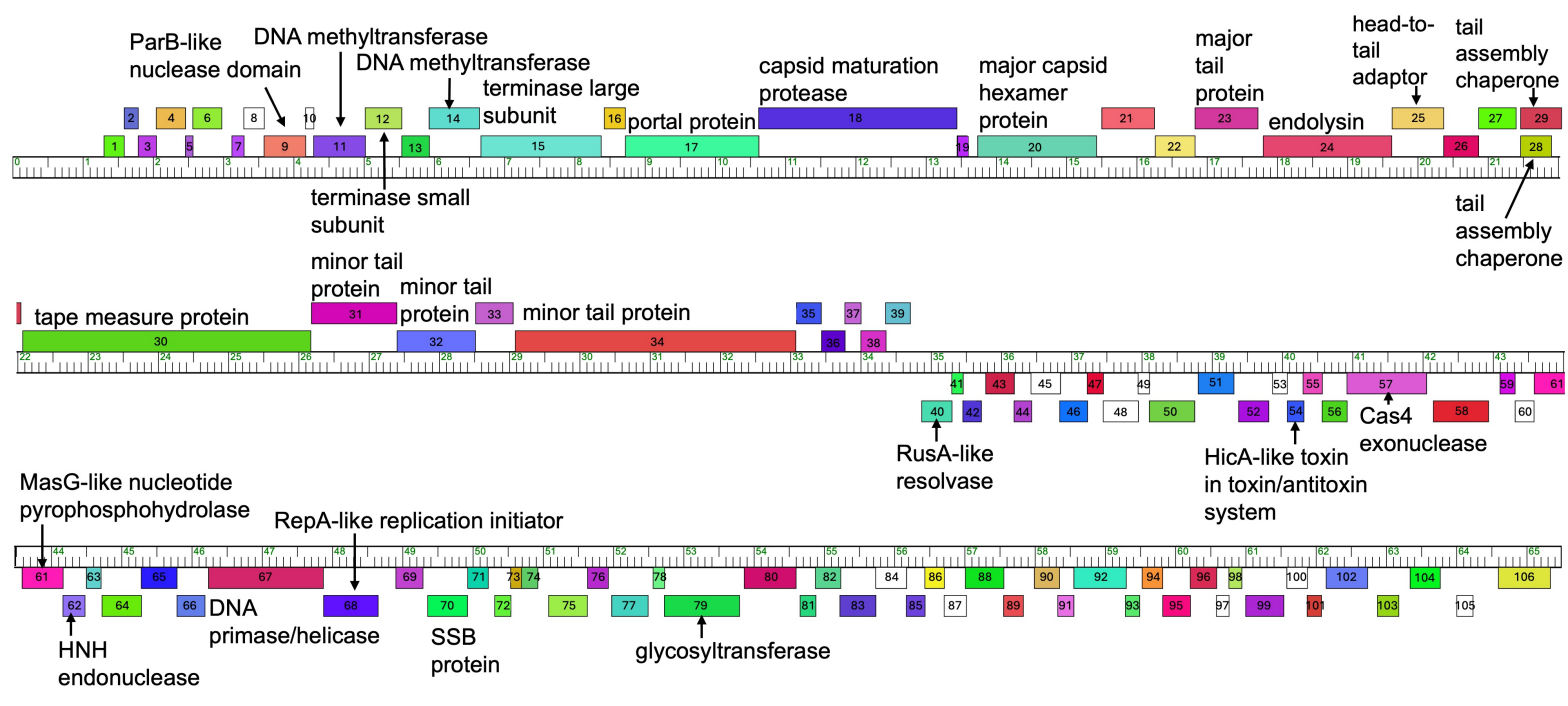
Each box indicates a putative gene, with the number inside indicating its gene number in the genome. The ruler indicates the base pair location within the genome, with each hatch mark representing 100 base pairs. Boxes above the ruler indicate putative genes are located on the forward strand, and boxes below the ruler indicate putative genes are located on the reverse strand. Maps were generated using Phamerator with database Actino_Draft_579 (Cresawn et al., 2011).

## Description

Bacteriophages have been co-evolving with bacteria for over 3 billion years. Characterizing new bacteriophages taps their enormous yet under-explored genetic diversity to advance our understanding of virus-host interactions and for the development of phage as therapeutics (Oromí-Bosch et al. 2023) .

Bacteriophage AWGoat was isolated from a soil sample collected in Charlotte, NC (GPS coordinates: 35.162419 N, 80.56909 W) in 2023, using *Arthrobacter globiformis* NRRL B-2880 as the host. For isolation, the soil sample was incubated in PYCa liquid medium (peptone, yeast extract, calcium) at 27°C and shaken for 1 hour at 250rpm. The soil sample was centrifuged at 2,000xg for 10 minutes to pellet the soil. The supernatant was filtered through a 0.22 um syringe filter. The filtrate was inoculated with *A. globiformis* and incubated at 27°C for two days. The resulting culture was centrifuged at 2,000xg for 10 minutes, and the supernatant refiltered. Plaques formed on lawns grown from plating 0.5 mL saturated *A. globiformis* culture and 10 µL of the serially-diluted filtrate in 4.5ml PYCa top agar on 10ml PYCa agar plates. Plates were incubated 2–5 days at 27°C. AWGoat formed small, clear, round plaques 1.1 - 2.0 mm (n=10) in diameter. AWGoat was purified through two rounds of plating.

Genomic DNA was isolated from phage lysate using the Promega Wizard DNA cleanup kit. The NEB Ultra II Library Kit was used for library preparation and the DNA was then sequenced by Illumina sequencing (v3 reagents). This resulted in 558,338 150-base single-end reads with 1226-fold coverage. The assembly and finishing of the genome were completed using Consed v29 (Gordon et al. 1998; Russell 2018) and Newbler v2.9 (Miller et al. 2010) . The genome length is 65,496 bp with a GC content of 67.1% and defined direct terminal repeat ends. Default parameters were used for all software.

Auto-annotation was performed in DNAMaster v2705 ( http://cobamide2.bio.pitt.edu ), which utilized Glimmer v3.02 (Delcher et al. 2007) and GeneMark v3.25 (Besemer and Borodovsky 2005) . Starts were manually evaluated using BLASTp v2.15.0 (non-redundant protein sequences) (Altschul et al. 1990) and Starterator v556 ( http://phages.wustl.edu/starterator/ ). Functions were manually assigned using DeepTMHMM v1.0.24 (Hallgren et al. 2022) , Phamerator v509 (Cresawn et al. 2011) , PECAAN v20240320 ( discover.kbrinsgd.org ), tRNAscanSE v2.0 (Lowe and Eddy 1997) , and HHPred using databases PDB_mmCIF70, SCOPe70, Pfam-A, and NCBI_Conserved_Domains CD (Söding 2005) . Default parameters were used for all programs. A total of 106 genes were predicted. No tRNAs were identified. Twelve orphams, putative genes with no homologues in the Actinobacteriophages database ( https://phagesdb.org/ ), were found within the genome. Nine membrane proteins were identified and 28 genes were assigned putative functions, including MazG-like nucleotide pyrophosphohydrolase, endolysin, and HNH endonuclease ( Figure 1 ).

AWGoat was assigned to cluster AP and subcluster AP4 based on the previously described criteria whereby gene content similarity (GCS) must exceed 35% to phages in the Actinobacteriophage database (Pope et al. 2017; Russell and Hatfull 2017) . To date, seventeen phages have been assigned to Cluster AP, and two phages have been assigned to subcluster AP4 (AWGoat and SilentRX). Similar to several other AP bacteriophages, the putative minor tail protein (AWGoat *34* ) is large, similar in size to the tape measure protein ( Figure 1 ; Klyczek et al. 2017). The putative endolysin gene, typically downstream of tape measure, is located upstream of the tape measure protein; however, this placement is common in AP cluster phages and has also been observed for *Arthrobacter* phages in other clusters (Klyczek et al., 2017) . A HicA-like toxin of the toxin/antitoxin system (gene *54* ) was also identified in AWGoat, homologues of which are found within several other AP cluster phages. Finally, no integrase or immunity repressor functions could be identified, consistent with other AP phages and suggesting that AWGoat is unlikely to establish lysogeny.

**Nucleotide sequence accession numbers**

AWGoat is available at Sequence Read Archive (SRA) No. SRX24123900. The GenBank Accession Number is PP978859.
